# Impact of quercetin, carnosine, and ozone in the cryopreservation on Nellore (*Bos indicus*) semen

**DOI:** 10.1590/1984-3143-AR2022-0048

**Published:** 2023-03-31

**Authors:** Willian Vaniel Alves dos Reis, Raiza Rocha Pereira, Mozarth Vieira, Cibele Cristina Tavares da Cunha, Bianca Rodrigues Acácio, Gustavo Guerino Macedo, Eliane Vianna da Costa-e-Silva, Breno Fernandes Barreto Sampaio

**Affiliations:** 1 Faculdade de Medicina Veterinária e Zootecnia, Universidade Federal de Mato Grosso do Sul, Campo Grande, MS, Brasil

**Keywords:** lipid peroxidation, reactive oxygen species, spermatozoa

## Abstract

The objective of this study was to reduce the effects of cryoinjury caused in bovine semen by cryopreservation. Ejaculates were collected from Nellore bulls and subjected to freezing in C (control), ozone (15, 30, and 60 µg mL^-1^ of ozone), quercetin (25, 50, and 100 µg mL^-1^ of quercetin), and carnosine groups (100, 200, and 300 ng mL^-1^ of carnosine). Samples were evaluated post-thaw (M0) and post-rapid thermoresistance test (M30) for sperm kinetics (total motility, progressive motility, curvilinear speed, linearity and amplitude of lateral head displacement) and cell structure viability (plasma membrane integrity, acrosomal integrity, mitochondrial potential, membrane fluidity, and lipid peroxidation). There were no differences (*P > 0.05*) between the control, quercetin, and carnosine-treated groups for the parameters evaluated at M0 and M30. In turn, supplementation with ozone resulted in lower values for sperm kinetics (*P < 0.05*) and lower mitochondrial potential at M30 (*P < 0.05*). Quercetin and carnosine at the concentrations used did not promote significant gains in frozen semen, nor did they demonstrate cytotoxicity. We expected to obtain positive results with the use of ozone. Nonetheless, the addition was harmful to the parameters of sperm kinetics, and its effect was not observed as a possible pro-antioxidant. We believe that the fact that the gas did not harm the sperm structure opens avenues for tests with lower dosages, since, by reducing its concentration, we could minimize the damage to sperm kinetics.

## Introduction

All thermal stresses owing to cryopreservation cause changes in the energy metabolism of the cell. When oxidative stress occurs, leading to an increase in reactive oxygen species (ROS), surpassing the quantity of antioxidants is primarily related to the reduction of sperm motility (Ruiz-Pesini et al., 1998). This process results in a considerable proportion of cells that do not resist, which can compromise its final use in the fertilization of the oocyte (Leite et al., 2011). It is estimated that frozen semen presents an approximately 50% decrease in sperm viability compared to fresh semen (Chacur et al., 2012).

Bovine ejaculate is naturally composed of antioxidants that neutralize ROS, such as superoxide dismutase, catalase, glutathione peroxidase, vitamin A, and vitamin E (Bollwein and Bittner, 2018; Patel et al., 2016; Tvrdá et al., 2013). However, due to the semen dilution process for cryopreservation, there is a reduction in the availability of these antioxidants, which can promote the accumulation of ROS (Andrade et al., 2010; Duque et al., 2017; Maia and Bicudo, 2009). Thus, the effect of supplementing dilution media with antioxidants to prepare, maintain, and cryopreserve sperm has been studied to reduce the effects caused by this imbalance (Mortimer, 2000). These antioxidants are classified as enzymatic or non-enzymatic, where enzymatic are composed of enzymes naturally produced by the organism (endogenous), while non-enzymatics are mainly acquired from nutritional sources (exogenous) (Barbosa et al., 2010).

Found in large quantities in fruits and vegetables and due to its high capacity for sequestration and neutralization of free radicals as a consequence of the hydroxyl groups belonging to its structure (Behling et al., 2004), Quercetin flavonoid has been studied as a potent non-enzymatic antioxidant (Silva et al., 2012). Its activity has already been described for several other functions, such as anti-tumor, anti-inflammatory, and antimicrobial, in addition to its antioxidant activity (Santos and Rodrigues, 2017). Moreover, quercetin's ability to maintain sperm viability has been successfully adopted in several species such as horses (Gibb et al., 2013; McNiven and Richardson, 2006), goats (Silva et al., 2016), cattle (Tironi et al., 2019), buffalo (Ahmed et al., 2019), wild pigs (Winn and Whitaker, 2018), and humans (Zribi et al., 2012), demonstrating promising results.

One of the end products of lipid peroxidation, resulting from the metabolism of reactive oxygen species, which is responsible for promoting the activation of pro-inflammatory cytokines, such as TNF-ß and IL-8, is malondialdehyde (Hipkiss et al., 1998). Found naturally in the mammalian organism, carnosine (β-alanyl-L-histidine) acts by eliminating the products responsible for lipid peroxidation, with greater influence on the neutralization of the malondialdehyde molecule, as pointed out by Drozak et al. (2010). As carnosine is a protein native to the organism and soluble in water, this facilitates dilution and allows incorporation into the diluent for cryopreservation without affecting its composition. Combining these factors with its power to neutralize free radicals, it is suggested that it can promote antioxidant and beneficial functions in cryopreserved semen.

Ozone gas (O^3^), although known to be a powerful oxidizer (Sunnen, 1988), has been challenged for a possible indirect antioxidant effect. If used safely, its oxidizing power induces mild oxidative stress, promoting the elevation of enzymatic antioxidants (Alves et al., 2004), thus causing a blockage of the generation of free radicals (Agrillo et al., 2006). However, its activity for this purpose has not yet been evaluated in sperm.

Owing to the facts presented, the present study aimed to evaluate the addition of the quercetin flavonoid, carnosine, or ozone gas to the freezing diluent, to reduce the cryoinjury caused to the bovine semen.

## Materials and methods

This study was approved by the Animal Use Ethics Committee (CEUA) of the Federal University of Mato Grosso do Sul, UFMS, Campo Grande - MS, under protocol No. 1.082/2019.

### Animals used

Five Nellore (*Bos indicus*) bulls, aged between 24 and 36 months, were kept in paddocks under the same environmental conditions, with mineral supplementation and water provided *ad libitum.* All animal were submitted to andrological evaluation 1 week before the beginning of the experiment and were approved in accordance with the parameters established by the Manual of Andrological Examination and Animal Semen Evaluation of the Brazilian College of Animal Reproduction (CBRA, 2013).

### Quercetin, carnosine, and ozone

Quercetin (Sigma-Aldrich – Q4951) was diluted in dimethyl sulfoxide (DMSO) to a final concentration of 0.1% after incorporation into semen. This concentration corresponds to the maximum non-harmful concentration of DMSO in the sperm (Meamar et al., 2012). Solid carnosine (Sigma-Aldrich – C9625), previously diluted in deionized water, was used.

Ozone was obtained using a portable ozone generator model O&L 1.5 (Ozone & Life™), programmed to release a flow of medicinal ozone (O_3_) sufficient to produce the concentrations indicated for the experimental groups. A 5 mL sterile syringe was attached to the device before starting the process, where 2.5 mL of O_3_ gas was stored immediately before contacting the semen. For O_3_ incorporation, 2.5 mL of semen was mixed with the gas inside the syringe for 30 s.

### Sample collection and cryopreservation

Three collections were carried out per bull (n = 15) by electroejaculation, with an interval of 1 week between each collection. Immediately after collection, ejaculate volume (mL), sperm concentration (×10^6^ sperm mL^-1^), motility (%), and vigor [1–5; CBRA (2013)] were measured. Only ejaculates that presented a minimum of 80% of motile sperm and vigor 3 were used in this experiment. The commercial diluent Botu-Bov™ (Botupharma, Botucatu, São Paulo, Brazil) was used.

Each ejaculate was distributed in 10 experimental groups, namely, the Control (without the addition of ozone, quercetin or carnosine), O15 (15 µg mL^-1^ of ozone), O30 (30 µg mL^-1^ of ozone), O60 (60 µg mL^-1^ of ozone), Q25 (25 µg mL^-1^ of quercetin), Q50 (50 µg mL^-1^ of quercetin), Q100 (100 µg mL^-1^ of quercetin), CAR100 (100 ng mL^-1^ of carnosine), CAR200 (200 ng mL^-1^ of carnosine), and CAR300 (300 ng mL^-1^ of carnosine) groups.

Each semen straw was packed with 30 million viable sperm cells. The semen was stored in 0.5 mL straws and subjected to the conventional cryopreservation process (Abud et al., 2014). Subsequently, they were stored in cryogenic cylinders until they were thawed for post-thaw evaluation.

### Thawing and post-thawing analyses

The semen was thawed in a water bath at 37°C for 30 s. The analyses were performed immediately post thaw (M0) and after the rapid thermoresistance test [RTT - incubation at 46°C for 30 min; M30; Vianna et al. (2009)].

Sperm kinetics analyses were performed using a computerized system (SCA^™^ - Sperm Class Analyzer, Microptic, Barcelona, Spain). The variables used were total motility (TM; %), progressive motility (PM; %), curvilinear velocity (VCL; μm s^-1^), the amplitude of lateral head displacement (ALH; μm), and linearity (LIN; %), that correspond to the kinetic characteristics most closely correlated with sperm hyperactivation in a semen sample (Nassar et al., 1998; Verstegen et al., 2002).

Plasma membrane integrity, acrosome integrity, plasma membrane fluidity, mitochondrial activity, and susceptibility to lipoperoxidation were assessed using flow cytometry. The supplemental data in terms of the fluorescent probes used are described in Table 1.

**Table 1:** Supplemental data about the fluorescent probes used to assess cellular structural viability via flow cytometry, with their respective interpretations.

**Rated parameter**	**Fluorescent probe**	**Reading**
Discard the non-cellular particles	Hoechst 33342 (H33342; 7 µM; Thermo Scientific, Fisher, IL, EUA)	Emits blue fluorescence when bound to DNA
Plasma membrane integrity	Propidium Iodide (PI; 1.5 µM; Sigma-Aldrich Co., Saint Louis, Missouri, EUA)	PI+ = Injured plasma membrane (Hossain et al., 2011).
Acrosomal membrane integrity	Peanut agglutinin conjugated to fluorescein isothiocyanate (FITC-PNA; 2ng; Sigma-Aldrich Co., Saint Louis, Missouri, EUA)	FITC-PNA+ = Reacted acrosomal membrane (Hossain et al., 2011).
Mitochondrial activity	MitoStatus Red (MST; 20 nM; BD PharmigenTM)	MST+ = High mitochondrial membrane potential (Carneiro et al., 2018)
Membrane fluidity	Merocyanine (MERO; M24571; 7.5 μM; Life Technologies) associated with the Yo-pro-1 probe (YO; Y3603; 25 nM; Life Technologies)	YO+ = Injured plasma membrane; MERO+ = lipid bilayer disorder (Harrison et al., 1996).
Susceptibility to lipoperoxidation	C11-BODYPY (PERO; D-3861; Molecular Probes)	PERO+ = indicates that cells are peroxidizing (Neild et al., 2005).

### Flow cytometry

Flow cytometry procedures were carried out with CytoFLEX^™^ (Beckman Coulter, Brea, California, USA) equipped with blue (488-nm, 100 mW), red (640-nm, 40 mW) and violet (405-nm, 100 mW) lasers and the data were analyzed using CytExpert Acquisition software (Beckman Coulter, Brea, California, USA). For the reading on the flow cytometer, all the semen samples were diluted to a concentration of 5 × 10^6^ sperm mL^-1^ in TALP-PVA buffer solution (100 mM NaCl, 3.1 mM KCl, 25.0 mM NaHCO3, 0.3 mM NaH2PO4, 21.6 mM DL 60% sodium lactate, 2.0 mM CaCl2, 0.4 mM MgCl2, 10.0 mM Hepes-free acid, 1.0 mM sodium pyruvate, 1.0 mg mL^-1^ polyvinyl alcohol-PVA and 25 μg mL^-1^ gentamicin, according to Carneiro et al. (2018), in a final volume of 200 µL, followed by 7 µM of Hoechst 33342 (H33342; Thermo Scientific, Fisher, IL, EUA – H1399). This combination was important to discard the non-cellular particles in the samples (Parrish et al., 1988). For each assay, at least 10,000 cells were evaluated by the flow cytometer for each sample. The data were generated in a histogram graph format, which allowed the visualization of all visible events, properly compensated by the flow cytometer software matrix itself.

#### Plasma membrane and acrosome integrity

For the evaluation of plasma membrane and acrosome integrity, we used the following fluorescent probes: propidium iodide (PI; Sigma-Aldrich Co., Saint Louis, Missouri, EUA – P4170), and peanut agglutinin conjugated to fluorescein isothiocyanate (FITC-PNA; Sigma-Aldrich Co., Saint Louis, Missouri, EUA L7381), according to Freitas-Dell’Aqua et al. (2012). We added to the aliquot 1.5 µM of PI, and 2 ng of FITC-PNA (of a 1 mg mL^-1^ solution), and the sample was incubated for 20 min at 37°C protected from light. At the end of this period, the sample was evaluated on a flow cytometer for analysis of florescence emission. Intact cells are not stained by the reagents. Spermatozoa were classified as intact plasma membranes with intact acrosomes (PIPNA; %).

#### Membrane fluidity

For the evaluation of membrane fluidity, was added 7.5 μM of the Merocyanine probe (MERO; M24571; Life Technologies) and 25 nM of the probe Yo-pro-1 (YO; Y3603; Life Technologies) in another sample already diluted with TALP-PVA and H33342 as in the previous process. The sample was incubated for 20 min at 37°C protected from light for later assessment by flow cytometry. Pannexin channels allow the flow of ions and larger molecules across the membrane, such as ATP (Dufresne and Cyr, 2014). When plasma membrane destabilization occurs by increasing the permeability of these channels, the YO is able to penetrate the cell, while the MERO detects changes in the arrangement of lipids contained in the membrane. All of this happens even before the total loss of integrity, as an initial modification of the enablement process. Therefore, the greater the permeability, the greater the binding of the dyes (Harrison et al., 1996). After, the sperm were classified into intact and non-fluid plasma membranes (YOMERO; %).

#### Mitochondrial potential

For the evaluation of mitochondrial potential, another already diluted sample was added of 20 nM of the MitoStatus Red fluorescent probe (MST; BD Pharmingen^™^), whose fluorescence emission indicates the high potential of the mitochondrial membrane (Camargo et al., 2017). The sample was incubated for 20 min at 37°C protected from light for later assessment by flow cytometry. In association with propidium iodide, sperm were classified into the intact plasma membrane and high mitochondrial potential (PIMST; %).

#### Lipid peroxidation (Hoechst and Bodipy)

For lipid peroxidation evaluation, we used C11-BODIPY (BODIPY; D-3861; Molecular Probes) according to the protocol described by Guasti et al. (2012). BODIPY (5 μM) was added to the sample diluted according to a previous procedure and then incubated at 37°C for 30 min. After incubation, two consecutive washes were performed by centrifugation at 300 G for 5 min with TALP-PVA, and the pellet was resuspended in 500 μL of TALP-PVA for reading on the flow cytometer. Once oxidized, BODIPY converts its fluorescence from red to green. The percentage of peroxidized cells was classified as PERO (%).

### Statistical analysis

The free software SAS™ for academics was used to perform the statistical analyzes. The analyzes were based on answering the following questions: Was there a treatment effect for each moment? And if so, where is the difference? The values were analyzed by Proc GLIMMIX, considering moment and concentrations of carnosine, quercetin and ozone. In case of significance, the adjusted Tukey test was used to test for differences among treatments. Significance was set at *P < 0.05*. All the data are depicted in tables with variables presented as means ± SEM to facilitate reader’s interpretation.

## Results

The control group expressed values ​​considered high for TM and PM post-thaw (CBRA, 2013), demonstrating that the bulls whose ejaculates were cryopreserved were good freezers and that the cryopreservation process was well conducted.

The mean values (± standard error) of kinetic sperm variables at post-thaw and post-RTT moments are identified in Tables 2-3.

**Table 2:** Mean values (± standard error) of kinetic sperm variables at post-thaw moment (M0).

**Post-Thaw – M0**											
**Sperm variable**	**CONTROL**	**CAR100**	**CAR200**	**CAR300**	**O15**	**O30**	**O60**	**Q25**	**Q50**	**Q100**	**p-value**
TM (%)	55.56 ± 26.45^a^	54.05 ± 20.67^a^	53.50 ± 18.98^a^	60.91 ± 23.14 ^a^	28.46 ± 21.82^b^	19.88 ± 12.76^b^	16.48 ± 10.41^b^	59.34 ± 22.61 ^a^	58.69 ± 19.79 ^a^	62.44 ± 24.27^a^	<0.0001
PM (%)	37.11 ± 22.36^a^	37.05 ± 18.35^a^	35.62 ± 15.89^a^	41.09 ± 19.31^a^	8.90 ± 18.39 ^b^	3.31 ± 6.43^b^	1.12 ± 1.87^b^	39.46 ± 19.85^a^	37.79 ± 17.85^a^	41.18 ± 19.02^a^	<0.0001
VCL (μm s-1)	52.16 ± 10.53^a^	52.44 ± 9.76^a^	51.56 ± 10.87^a^	53.09 ± 11.54^a^	23.44 ± 12.27^b^	19.51 ± 7.15^b^	17.20 ± 4.46^b^	54.18 ± 12.31^a^	53.98 ± 11.93^a^	54.76 ± 10.72^a^	<0.0001
LIN (%)	59.25 ± 4.31^a^	59.53 ± 7.11^a^	58.24 ± 8.16^a^	58.89 ± 9.19^a^	30.67 ± 13.03^b^	26.50 ± 11.06^bc^	19.86 ± 7.83^c^	61.70 ± 8.68^a^	59.99 ± 8.88^a^	59.52 ± 8.86^a^	<0.0001
ALH (μm)	2.20 ± 0.21^a^	2.20 ± 0.18^a^	2.29 ± 0.36^a^	2.21 ± 0.18^a^	1.39 ± 0.68^b^	0.90 ± 0.77^bc^	0.68 ± 0.70^c^	2.21 ± 0.14^a^	2.27 ± 0.29^a^	2.28 ± 0.31^a^	<0.0001

TM: total motility; PM: progressive motility; VCL: curvilinear velocity; LIN: linearity; ALH: amplitude of lateral head displacement; CONTROL: without the addition of ozone, quercetin or carnosine; CAR100: 100 ng mL-1 of carnosine; CAR200: 200 ng mL-1 of carnosine; CAR300: 300 ng mL-1 of carnosine; O15: 15 µg mL-1 of ozone; O30: 30 µg mL-1 of ozone; O60: 60 µg mL-1 of ozone; Q25: 25 µg mL-1 of quercetin; Q50: 50 µg mL-1 of quercetin; Q100: 100 µg mL-1 of quercetin. Different letters on the line indicate difference between means by the Tukey test (P < 0.05).

**Table 3:** Mean values (± standard error) of kinetic sperm variables at post-Rapid Thermoresistance Test moment (RTT – M30).

**Post-Thaw – M30**											
**Sperm variable**	**CONTROL**	**CAR100**	**CAR200**	**CAR300**	**O15**	**O30**	**O60**	**Q25**	**Q50**	**Q100**	**p-value**
TM (%)	30.42 ± 20.95^a^	29.10 ± 18.55^a^	31.61 ± 19.54^a^	31.09 ± 17.35^a^	14.82 ± 15.52^ab^	7.21 ± 7.61^b^	7.22 ± 4.28^b^	33.89 ± 20.61^a^	30.91 ± 19.49^a^	29.41 ± 22.27^a^	<0.0001
PM (%)	18.59 ± 15.46^ab^	17.56 ± 15.52^ab^	18.70 ± 14.92^ab^	18.17 ± 13.11^ab^	3.13 ± 11.13^bc^	0.31 ± 086^c^	0.16 ± 0.25^c^	19.57 ± 16.74^a^	17.08 ± 15.20^ab^	17.06 ± 16.45^ab^	<0.0001
VCL (μm s-1)	37.49 ± 13.58^a^	38.72 ± 16.07^a^	38.21 ± 16.73^a^	41.15 ± 13.79^a^	18.12 ± 10.28^b^	14.42 ± 4.35^b^	15.14 ± 2.36^b^	40.27 ± 13.73^a^	37.37 ± 11.98^a^	38.48 ± 14.15^a^	<0.0001
LIN (%)	48.96 ± 21.17^a^	52.08 ± 21.92^a^	47.68 ± 18.71^a^	53.72 ± 20.48^a^	16.64 ± 15.40^b^	15.68 ± 7.40^b^	13.00 ± 4.14^b^	52.70 ± 15.24^a^	51.51 ± 15.95^a^	49.75 ± 18.52^a^	<0.0001
ALH (μm)	1.83 ± 0.69^a^	1.79 ± 0.52^a^	1.89 ± 0.45^a^	1.78 ± 0.49^a^	0.45 ± 0.66^b^	0.29 ± 0.63^b^	0.23 ± 0.48^b^	1.92 ± 0.65^a^	1.86 ± 0.53^a^	1.94 ± 0.63^a^	<0.0001

TM: total motility; PM: progressive motility; VCL: curvilinear velocity; LIN: linearity; ALH: amplitude of lateral head displacement; CONTROL: without the addition of ozone, quercetin or carnosine; CAR100: 100 ng mL-1 of carnosine; CAR200: 200 ng mL-1 of carnosine; CAR300: 300 ng mL-1 of carnosine; O15: 15 µg mL-1 of ozone; O30: 30 µg mL-1 of ozone; O60: 60 µg mL-1 of ozone; Q25: 25 µg mL-1 of quercetin; Q50: 50 µg mL-1 of quercetin; Q100: 100 µg mL-1 of quercetin. Different letters on the line indicate difference between means by the Tukey test (P < 0.05).

When compared to the control group, there was no improvement in the analyzed kinetic variables (*P > 0.05*) as an effect of supplementation with quercetin and carnosine.

The results for TM and PM at moments 0 and 30 did not show any significant difference between the quercetin, carnosine, and control treatments (*P > 0.05*); however, they showed differences when compared to the treatments with ozone (*P < 0.0001*), with lower values resulting from ozone treatments. Ozone reduced the values ​​for the same variables at both M0 and M30.

At TM-M0, we observed that all treatments using carnosine were similar to the control group (P > 0.05). Therefore, although we have not been successful, the use of carnosine is not harmful to spermatozoa at the concentrations used, supporting further investigations. Our study found similarities between control, quercetin, and carnosine treatments with respect to variables VCL, LIN, and ALH.

**Figure 1:**
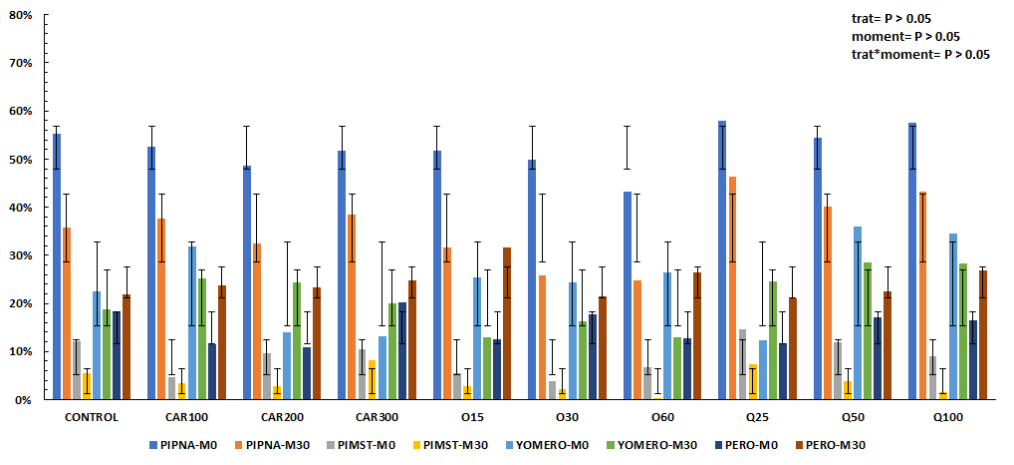
Flow cytometry results (values expressed in percentage) at post-thaw (M0) and post-RTT (M30) moments. PIPNA: intact plasma membranes with intact acrosome; PIMST: intact plasma membrane and high mitochondrial potential; YOMERO: intact and non-fluid plasma membrane; PERO: percentage of peroxidized cells; CONTROL: without the addition of ozone, quercetin or carnosine; CAR100: 100 ng mL-1 of carnosine; CAR200: 200 ng mL-1 of carnosine; CAR300: 300 ng mL-1 of carnosine; O15: 15 µg mL-1 of ozone; O30: 30 µg mL-1 of ozone; O60: 60 µg mL-1 of ozone; Q25: 25 µg mL-1 of quercetin; Q50: 50 µg mL-1 of quercetin; Q100: 100 µg mL-1 of quercetin.

There were no significant differences between treatments for flow cytometry results (Figure 1; *P > 0.05*). No higher mitochondrial potential (PIMST; *P > 0.05*) was observed in the quercetin and carnosine groups compared to the control group, corroborating similar results in TM and PM. If there was greater mitochondrial potential, this could, as a consequence, increase the motility of sperm cells. However, it cannot be said that the addition of quercetin and carnosine was harmful to the sperm cell at the concentrations used. Also, for the variables PIPNA, YOMERO and PERO, it was not possible to identify significant differences between the groups (*P > 0.05*) in both moments, as shown in Figure 1.

Comparing to carnosine, quercetin and control groups, the sperm kinetic variables were decreased when semen was cryopreserved with ozone, irrespectively of the concentration used, and that probably low values ​​for TM and PM were reflected in the other low values.

## Discussion

Motility is one of the first functions affected by ROS due to oxidative stress (Aitken et al., 2014). Quercetin and carnosine showed no signs of hyperactivation in their samples. However, the samples of the control group remained of quality in the same evaluations. These results lead us to believe that, although the findings are significantly the same, the fact that the animals used were good semen freezers masked the results and that quercetin and carnosine would probably have a better action if the semen analyzed were from low performance upon cryopreservation animals, offering a greater challenge for the antioxidant in question.

Ozone TM and PM values showed that at these concentrations, its oxidizing power (Sunnen, 1988) probably prevailed as the concentrations used were not low enough for ozone to express its pro-antioxidant factor (Alves et al., 2004), that is, its ability to stimulate the production of antioxidants, which, if it occurs, would make it biologically useful for such practice.

Testing the supplementation of 25, 50, 100, and 200 µg mL^-1^ of quercetin in the cryopreservation of Dutch bull semen, Avdatek et al. (2018) found that except for the lowest concentration (50 μg mL^-1^), upon thawing, treatment with quercetin resulted in lower TM and PM, and the other kinetic characteristics worsened as the dosage of the antioxidant in question increased, to the point of being significantly lower than the control group (*P < 0.05*) in the two highest concentrations tested. This divergence between our results and those of the authors in question with the use of the same concentrations could be correlated with the vehicle used to dilute quercetin, since our vehicle was DMSO, offering less aggression to the cell than ethanol (Timm et al., 2013), adopted by them.

In an experiment conducted by Rocha et al. (2018) in semen stallions, groups with higher concentrations of carnosine were better freezers than those with lower concentrations. Considering the differences between species, no improvement of sperm survival after freezing was found - this is in contrast to the positive impact of carnosine in stallion semen.

In an experiment with Brahman bulls, Tironi et al. (2019) tested lower concentrations (5, 15, and 20 μg mL^-1^), and although they observed an increase in TM and PM values ​​in semen supplemented with quercetin, they also found no significance in their results for the doses tested. However, the same authors found that the LIN values ​​increased as the dose of the antioxidant increased, being higher (*P < 0.05*) than the control at the highest concentration of quercetin. The authors in question draw attention to the fact that the control group did not have a high percentage of the total (7.4%) and progressive (5.0%) motility, indicating that quercetin acted by improving the linearity index in samples with inferior quality.

Diverging from our results, when submitting dog semen to different concentrations of carnosine, Losano et al. (2017) found that the treatment had a negative effect on motility, as the dosage was increased, suggesting that carnosine may influence the production of ATP, suggesting that this dipeptide has a direct influence on the inhibition of glycolysis, decreasing the production of ATP (Beorlegui et al., 1997; Hipkiss and Gaunitz, 2014).

The same concentrations of quercetin were used by Avdatek et al. (2018) in cattle and the doses of 0.2 and 0.3 mM were tested in the equine specie by Seifi-Jamadi et al. (2016). For the first author, the use of quercetin as an antioxidant did not produce better results with regard to progressive and total sperm motility and plasma membrane integrity, agreeing with our results. However, there was a reduction in lipid peroxidation for the second reported author. It is worth mentioning that the doses used by (Seifi-Jamadi et al., 2016) are lower than those used by our group. Therefore, there are indications that there may be, in addition to interaction with the vehicle used, an interaction of the animal species with respect to the action of the antioxidant, since quercetin in horses improves some aspects while worsening others.

Studies related to supplementation of semen with ozone are scarce, which makes it challenging to choose the optimal dose that would meet the pro-antioxidant factor of that gas (Merhi et al., 2018). The results suggest that, although sperm kinetic values were impaired with ozone supplementation, it did not completely damage the cell structure, since there was no significant difference in PIPNA (intact plasma membrane and intact acrosomal membrane), YOMERO (intact membrane and organized bilayer), and PERO (percentage of peroxidized cells). This result encouraged us to consider the effect of ozone if its dosage was lower since for kinetics the values worsened with increasing dose and for the structure, there was no such damage.

## Conclusions

From the exposed results, it was possible to conclude that quercetin and carnosine at the concentrations used did not promote significant gains in frozen semen, nor did they demonstrate cytotoxicity. However, it was observed that ozone at the adopted concentrations did not promote improvement in the parameters of sperm kinetics, and its paradoxical effect as an antioxidant for these characteristics was not observed. However, since the gas did not damage the sperm cell structure, this does not prevent its use, and smaller doses must be tested for this purpose.
